# Self-employment And Cognitive Function Among Older Individuals: Evidence From The Health and Retirement Study

**DOI:** 10.1093/geroni/igaf122.2346

**Published:** 2025-12-31

**Authors:** Kingsley Mbam, Yan-Jhu Su

**Affiliations:** University of Massachusetts Boston, Boston, Massachusetts, United States; The Pennsylvania State University, University Park, Massachusetts, United States

## Abstract

A large body of research has shown that employment is related to cognitive functioning. However, how self-employment impacts cognition among older individuals is largely unaddressed. This study looks to address this gap by examining whether cognitive function changes over time among self-employed older individuals. This longitudinal study used data from 1998 to 2016 waves of the Health and Retirement Study (HRS) for participants who completed at least two waves and provided valid responses to the cognitive function scale (N = 19,242). Participants had 1998, 2004, and 2010 as baseline waves and were followed from these baseline waves until the 2016 wave. Cognitive function was a time-variant variable measured at the baseline and over time and was accessed with the modified version of the Telephone Interview for Cognitive Status (TICS). Self-employment was a time-invariant variable measured at the baseline only, which differed for participants depending on the wave they entered the study. Linear mixed-effects models were utilized to examine the impact of self-employment on cognitive function at the baseline and over time. Adjusted models results show that self-employed participants had higher cognitive function scores (b = 0.63, p = 0.001) than not self-employed participants at the baseline wave(s). The results also revealed that the interaction term between self-employment and time was statistically significant (b = -0.07, p < 0.001), suggesting variation in cognitive decline rate between self-employed individuals and not self-employed individuals. Strengthening existing socioeconomic policies to protect self-employed older individuals will ensure they remain cognitively active with self-employment for improved cognitive health.

